# Evidence for a Time-Invariant Phase Variable in Human Ankle Control

**DOI:** 10.1371/journal.pone.0089163

**Published:** 2014-02-18

**Authors:** Robert D. Gregg, Elliott J. Rouse, Levi J. Hargrove, Jonathon W. Sensinger

**Affiliations:** 1 Departments of Mechanical Engineering and Bioengineering, University of Texas at Dallas, Richardson, Texas, United States of America; 2 Department of Media Arts and Sciences, Massachusetts Institute of Technology, Cambridge, Massachusetts, United States of America; 3 Center for Bionic Medicine, Rehabilitation Institute of Chicago, Chicago, Illinois, United States of America; 4 Department of Physical Medicine and Rehabilitation, Northwestern University, Chicago, Illinois, United States of America; 5 Institute of Biomedical Engineering and Department of Electrical and Computer Engineering, University of New Brunswick, Fredericton, New Brunswick, Canada; Tokai University, Japan

## Abstract

Human locomotion is a rhythmic task in which patterns of muscle activity are modulated by state-dependent feedback to accommodate perturbations. Two popular theories have been proposed for the underlying embodiment of phase in the human pattern generator: a *time-dependent* internal representation or a *time-invariant* feedback representation (i.e., reflex mechanisms). In either case the neuromuscular system must update or represent the phase of locomotor patterns based on the system state, which can include measurements of hundreds of variables. However, a much simpler representation of phase has emerged in recent designs for legged robots, which control joint patterns as functions of a single monotonic mechanical variable, termed a *phase variable*. We propose that human joint patterns may similarly depend on a physical phase variable, specifically the heel-to-toe movement of the Center of Pressure under the foot. We found that when the ankle is unexpectedly rotated to a position it would have encountered later in the step, the Center of Pressure also shifts forward to the corresponding later position, and the remaining portion of the gait pattern ensues. This phase shift suggests that the progression of the stance ankle is controlled by a biomechanical phase variable, motivating future investigations of phase variables in human locomotor control.

## Introduction

Imagine walking along a riverbed and stepping on a rock that slips beneath your heel. This unexpected change in the ground slope rotates your foot towards your shank, i.e., dorsiflexes your ankle. If your ankle pattern was controlled as a function of time, your ankle would continue the normal pattern on top of this perturbation. However, your ankle normally dorsiflexes later in the walking gait (i.e., it would have encountered the dorsiflexed position anyway), so the response of minimal intervention [1] would be to continue from the later position and follow the remaining portion of the normal pattern. This shift in phase–or location in the gait cycle–highlights the difference in controlling locomotor patterns with respect to the current state rather than the current time.

For decades it has been known that mammalian locomotion employs state-dependent feedback to make phase corrections in response to external perturbations or sensory stimuli (see [2] for a review). Certain sources of state-dependent feedback appear to 1) gate discrete control events, such as the initiation of swing based on the hip angle [3], [4] and unloading of leg muscles [5], [6], and 2) modulate the timing of continuous behaviors [2], e.g., ankle-foot muscle and cutaneous afferents are used for active balance control [7]. These behaviors could be the result of state-dependent feedback modulating an internal timing variable that produces feedforward patterns of muscle activation [8], but it is also possible for reflex mechanisms and mechanical self-stabilization to generate locomotor patterns *without* a time-dependent feedforward contribution [9]–[11]. The key difference is whether the underlying representation of phase in the human locomotor pattern is *time-dependent* or *time-invariant*.

The question of time invariance in motor control has intrigued the robotics community from its earliest days. Although research in robotic pattern generation began with time-dependent control strategies (e.g., prosthetic echo control [12] and the standard tracking methods in [13]), the advantages of adjusting the phase of patterns based on state-dependent feedback were quickly realized, particularly with regard to energetics and stability. By the 1990s control strategies without any dependence on time were being used for robotic activities including hopping [14], juggling [15], and walking [16]. The phase of these patterns has typically been represented by the system state (as in [17], [18] for insect locomotion), but this can involve measurements of hundreds of state variables in complex systems like humans. Fortunately, a much simpler time-invariant representation of phase has emerged in recent designs for bipedal robots [19]–[22] and prosthetic legs [23]–[26], which control joint patterns as functions of a monotonic (e.g., strictly increasing) mechanical variable that uniquely represents the body’s progression through the gait cycle [27]. In other words, the progression of joint patterns is driven solely by measurements of a single physical variable (e.g., hip position), termed the *phase variable*.

**Figure 1:**
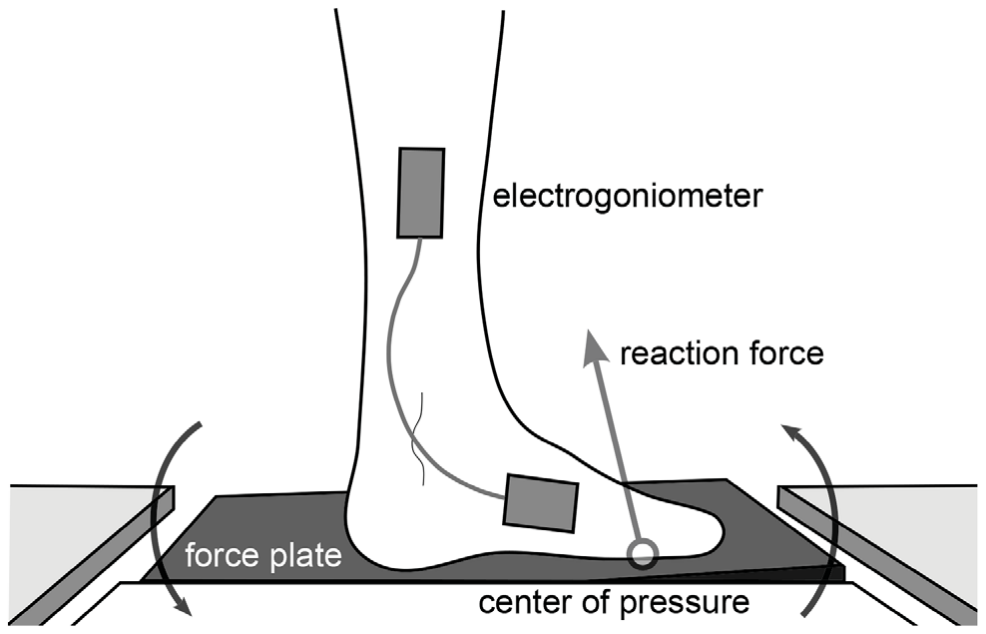
Diagram of robotic force plate dorsiflexing the stance foot. The platform’s center of rotation coincides with the ankle joint. The center of pressure (COP) is a candidate phase variable that can be measured by the force plate of the perturbation mechanism.

If human locomotion was similarly controlled with a phase variable, your neuromuscular system would sense a shift in the variable caused by a slope change and your ankle would respond according to the new phase location. We postulate that human locomotion is in fact controlled in this time-invariant manner, employing feedback related to a single state variable as a continuous representation of phase. Given substantial evidence that load-related receptors are involved in the initiation of phase-specific behaviors during human locomotion [5],[28]–[30], we hypothesize that human joint patterns depend on the heel-to-toe movement of the Center of Pressure (COP)–the point on the plantar sole of the foot (Figure 1) where the resultant reaction force is imparted against the ground, i.e., where all moments sum to zero [31]–[33].

**Figure 2:**
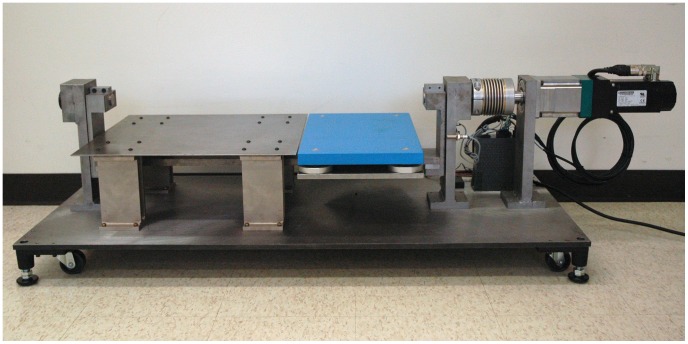
Perturbation mechanism before placement in elevated walkway.

The presence of a phase variable in human locomotor control can be tested experimentally with perturbations, because different motor control theories would present distinct transient responses in both the phase and time domains. Joint patterns look almost identical whether examined as functions of time or as functions of a phase variable during steady walking–when time and phase have a one-to-one relationship. However, a perturbation can decouple phase from time by causing a shift in the phase variable but not in time. This study analyzes the transient behavior of thirteen able-bodied human subjects walking over a robotic platform (Figure 1 and Figure 2) that unexpectedly changed the ground slope beneath the stance foot (displacing the ankle angle) with different timings, directions, and magnitudes. Beginning with a dataset of nine subjects perturbed by 2-degree rotations from [34] (which examined ankle impedance), we repeated the experiment with 5-degree perturbations on seven subjects (three repeats) to test the phase variable hypothesis across perturbation magnitudes. Although joint measurements were limited to the ankle, this is the primary joint moved by slope-changing perturbations [34], [35] and one of the primary joints responsible for adaptation to uphill walking [36]–[39]. This study employs a novel method for phase-domain analysis of the perturbation responses, wherein averages across trials are taken at points in phase (i.e., COP samples) rather than points in time. Our analysis found that ankle responses resemble patterns produced with a time-invariant phase variable, possibly related to the COP.

## Methods

### Ethics Statement

Thirteen able-bodied subjects gave written informed consent in accordance with study protocol STU00043310 approved by the Northwestern University Institutional Review Board.

### Hypotheses

Because we started with the 2-degree perturbation dataset from [34], which was limited to COP and ankle measurements, we focused our hypotheses on the response of the ankle joint. The ankle is one of the primary joints responsible for adaptation to uphill walking [36]–[39], and rotational perturbations move the ankle angle by the amount of the slope change [34], [35], see Video S1. We therefore considered how the human ankle adapts to inclined slopes in order to form hypotheses for a phase-based response of the ankle angle to rotational perturbations. In one pertinent study [37], human subjects walked on a level surface approaching a brief uphill incline, took a single step on the incline, and continued walking on a (raised) level surface beyond the incline. This study found that humans adjust to new terrain on the first step, where inclines less than 15 degrees result in an ankle trajectory that is dorsiflexed during stance by a nearly constant angle approximately equal to the slope. Similarly, the COP trajectory in a shank-based reference frame is rotated proportional to the slope angle during uphill walking [36], implying the ankle is dorsiflexed relative to the COP trajectory. Because the opposite is not true for downhill walking (i.e., the ankle is not plantarflexed by a downhill slope angle [36], [39]–[41]), this section focuses on the hypothetical responses to dorsiflexive perturbations and later considers the case of plantarflexive perturbations.

**Figure 3:**
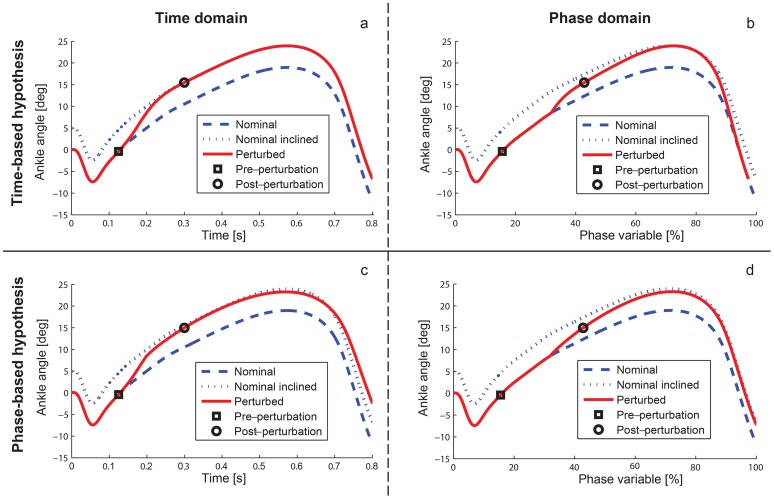
Hypothetical reference patterns in response to dorsiflexive (positive) perturbations at 100 ms after heel strike. Hypothetical reference patterns after 5-degree perturbation with time-based control (a, b) and phase-based control (c, d). The time domain (left) is normalized between heel strike and toe off, whereas the phase domain (right) is expressed over a hypothetical phase variable (Figure 4a). A phase variable would co-vary with the perturbation in such a way that the ankle state moves tangential to the nominal trajectory in phase domain, until the ankle adapts to the new ground slope (modeled at 75 ms after perturbation onset, see Methods) by converging to the offset trajectory. The phase domain (b) was computed from the assumed time-based reference pattern (a), whereas the converse was done from the phase-based reference pattern in (c–d), see Methods.

A dorsiflexive perturbation results in an uphill slope for the remainder of the single-support period, so we predicted that the perturbed ankle angle would begin to converge to a trajectory determined by the new slope angle after some adaptation delay (dotted curve in Figure 3a). Depending on the underlying control strategy, this convergence could take place as a function of a mechanical phase variable (e.g., Figure 3d), as a function of an internal timing variable (e.g., Figure 3a), or something in between these two hypotheses. These two hypotheses are opposing extremes in the spectrum of motor control possibilities, where some theories in between these extremes are described in [17]. It is generally accepted that human locomotion exhibits phase resetting behavior after perturbations [2], [8], [28], which is not reflected in the strict time-based control hypothesis of Figure 3a (i.e., a time-based response would resemble this hypothesis only until a phase reset occurred). We show this strict time-based hypothesis for the sake of comparison with the novel hypothesis we propose in this paper: control based on a physical phase variable.

Rotational perturbation experiments offer insight into the underlying control strategy of the human ankle, because different control strategies would result in different transient responses (i.e., convergence in the phase domain vs. the time domain) immediately after the perturbation. This observance of output data (the transient ankle trajectory) vs. input data (the perturbation slope) is the basis behind system identification theory [42]. In particular, we are most interested in the transient behavior of the ankle within the perturbed step, when the uphill slope condition holds. We can test the two control strategy hypotheses by inspecting this transient response for convergence in either the time domain or phase domain. If we were to observe the ankle response over multiple steps as the subject returned to steady-state walking, the phase- and time-domain representations would eventually become equivalent for both control hypotheses due to phase resetting.

In order to formulate testable sub-hypotheses, we derived reference ankle patterns, i.e., signals from a hypothetical pattern generator, for both the time-based and phase-based control strategies (Figure 3a–d). Although these idealized patterns do not reflect the dynamics of the ankle joint or perturbation mechanism, they offer templates for strategy-specific behaviors that might be found in the human transient response. We obtained the time-based reference pattern by first modeling the time domain (Figure 3a), where the nominal ankle trajectory was obtained from previously recorded able-bodied data, the inclined nominal trajectory was obtained by adding the final slope angle to the nominal ankle trajectory [36], [37], and the perturbed ankle trajectory was obtained by adding the (continuous) slope trajectory to the nominal ankle trajectory. For the phase-based pattern we first modeled the phase domain (Figure 3d), where the perturbed trajectory was obtained by adding an adaptation term to the nominal ankle trajectory. This adaptation effect was modeled at 75 ms after perturbation onset (the approximate latency observed in a similar perturbation study [43]) by an exponentially convergent trajectory going from zero toward the final slope angle. Other adaptation latencies and convergence rates are examined in Discussion, showing that the reference patterns are fairly insensitive to these assumptions.

**Figure 4:**
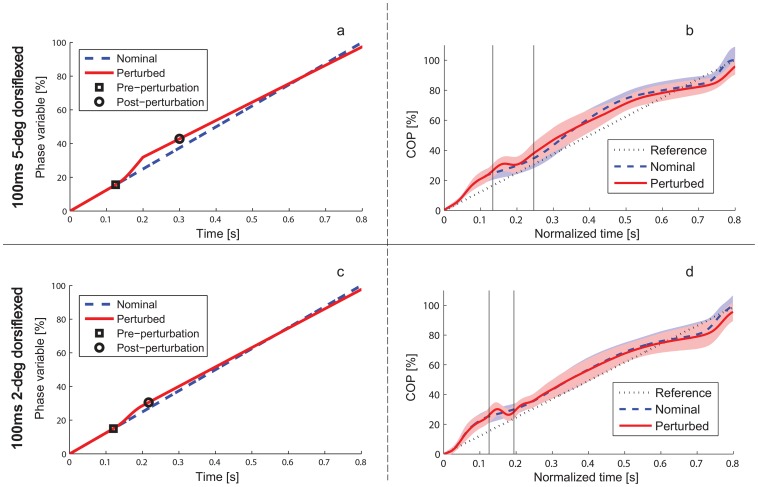
Hypothetical and human time-phase relationships. Left: Time-trajectory of hypothetical phase variable φ during nominal gait (dashed) and when subjected to 5-degree (a) and 2-degree (c) dorsiflexive perturbations (solid), where 0% and 100% respectively denote ipsilateral heel strike and toe off. Adaptation to the inclined slope is modeled as a shallower time-phase relationship starting at 75 ms after perturbation onset (see Methods for justification). Right: mean value and error bars (±1 standard deviation in shaded region) of the COP (our phase variable candidate) over time for the nominal condition (dashed) and 100 ms dorsiflexion condition (solid) from 5-degree experiment (b) and 2-degree experiment (d) in comparison with the reference phaseline 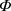 (dotted) used in the hypothetical time-phase relationship (left). In the nominal case the phase end-points 0% and 100% respectively correspond to mean COP values of 0 cm and 21.8 cm from the heel. The perturbation window is indicated by vertical lines. Note that the COP oscillates during the perturbation window because of the ramp-up acceleration and deceleration periods (see Methods). The human COP curve resembles the hypothetical (linear) time-phase relationship, but this phase variable could be better modeled with a nonlinear curve.

We then defined the hypothetical relationship φ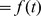 between time *t* and a hypothetical phase variable φ in Figure 4a. Because humans exhibit longer step durations during uphill walking [44] (and our experiments confirm that dorsiflexive perturbations cause longer step periods, see Results), we modeled the perturbation response with a shallower time-phase curve starting at 75 ms after perturbation onset [43]. This change in the phase trajectory after perturbations could be an adaptation effect or an intrinsic response due to a mechanical property of the phase variable (see Discussion). Given the time-phase relationship in Figure 4a and the time-based reference pattern in Figure 3a, we computed the corresponding phase-ankle relationship in Figure 3b by re-parameterizing the ankle trajectory from a function of time to a function of the hypothetical phase variable using nearest-neighbor interpolation in MATLAB. Conversely, we used the same time-phase relationship to compute the time domain (Figure 3c) from the phase-based reference pattern (Figure 3d). Note that the instantaneous tangential shift along the nominal trajectory in phase follows directly from this re-parameterization, i.e., it is only assumed in so far as Figure 3d and Figure 4a are assumed.

Given evidence that load-related receptors in the ankle-foot complex are involved in the initiation of phase-specific behaviors [5], [28]–[30], we hypothesized that the COP, which is local to the ankle-foot complex, might act as a phase variable for the ankle (reasons for and against this particular variable are examined in Discussion). We tested our phase variable hypothesis by examining ankle and COP responses to rotational perturbations (specifically dorsiflexion) at 100 ms, 225 ms, and 350 ms after initial heel strike (so sufficient time remained in the step for adaptation to the slope change). A phase-based response to a dorsiflexive perturbation would approximately match the hypothetical plots of Figure 3c–d, whereas a time-based response would be closer to Figure 3a–b until a phase reset occurred. In the case of phase-based control, the response to a dorsiflexive perturbation would converge in the phase domain toward the nominal trajectory offset by the perturbation angle (Figure 3d), and the response in time domain would initially undershoot and finally overshoot this offset trajectory (Figure 3c, as predicted by the transformation from phase to time domain). We evaluated this hypothesis for dorsiflexive perturbations in both the 2-degree (*n* = 9) and 5-degree (*n* = 7) experiments by performing point-wise inter-subject statistical tests (specifically the two-sided Student’s t-test) on the following sub-hypotheses:

- **H0.** The dorsiflexed step period is greater than the nominal step period (and the plantarflexed step period is less than the nominal step period).
- **H1.** For dorsiflexive perturbations the temporal ankle deviation (perturbed minus nominal angle in time domain) is less than the incline angle at time-points near peak dorsiflexion.
- **H2.** For dorsiflexive perturbations the temporal ankle deviation (perturbed minus nominal angle in time domain) is greater than the incline angle by the end of the timeline.
- **H3.** For dorsiflexive perturbations the phasic ankle deviation (perturbed minus nominal angle in phase domain) is not different than the incline angle by the end of the phaseline.

These parametric statistical tests used an alpha level of 0.05 (see Statistics below).

We also examined plantarflexive perturbations, but because humans do not normally plantarflex their ankle pattern by the slope angle during downhill walking [36], [39]–[41], we did not expect the ankle deviation to converge toward the perturbation incline angle in either domain. The stance ankle trajectory during downhill walking closely resembles (only slightly plantarflexed) the level-ground trajectory [39]–[41]. Therefore, in the case of phase-based control, the ankle response to a plantarflexive perturbation would converge in the phase domain to a trajectory in between the nominal ankle trajectory and the nominal trajectory offset by the perturbation angle. We applied the same inter-subject statistical test on the following sub-hypothesis:

- **H4.** For plantarflexive perturbations the phasic ankle deviation (perturbed minus nominal angle in phase domain) is less than zero and greater than the incline angle by the end of the phaseline.

In other words, if the ankle response remains bounded between the nominal trajectory and the slope-offset trajectory in the phase domain (rather than the time domain), this would suggest that the ankle tracked a phase-based reference trajectory. We now discuss the experiments used to test these sub-hypotheses.

### Experiments

The perturbation mechanism (Figure 2) had a 1.25 kW brushless AC motor (AKM-42H, Kollmorgen, Virginia, USA) with a 70∶1 transmission and a joint encoder (resolution: 3×10^−7^ radians), by which a PIC32 microcontroller and servodrive (AKD-B00606, Kollmorgen, Virginia, USA) tracked a pre-defined slope trajectory to produce perturbations. This trajectory had positive, zero, and negative periods of constant acceleration during a ramp-up window of 100 ms for 2 degrees and 125 ms for 5 degrees (which required an additional 25 ms due to hardware limitations). A force plate (9260AA3, Kistler, Winterthur, Switzerland) was mounted on top of the perturbation device [34], [35], Figure 1 and Figure 2, to measure the forces exerted by the stance foot. Force measurements during platform rotations were corrected for inertial forces due to the mass of the force plate (see [34]). Subjects were instrumented with an electrogoniometer (Delsys, Massachusetts, USA) that measured the ankle angle (resolution: 1×10^−4^ radians). Data were recorded synchronously with a 1 kHz sampling rate.

Able-bodied subjects were included if aged between 18 to 70 years and excluded if any of the following criteria were met: a body weight over 250 pounds, pregnant, a history of back and/or leg injury, joint problems, neuromuscular disorders, or any other impairment that could interfere with the study. Subjects were provided a ceiling-mounted harness and handrails to protect them from falls. The harness did not provide body-weight support, and the subjects were instructed not to use the handrails unless they lost balance (which very rarely occurred).

The perturbation device was placed within an elevated walkway to make a level walking surface. Each trial consisted of the subject walking along the walkway, stepping on the robotic force plate, walking a few more steps on the walkway and then stopping (Video S1). Subjects were asked to walk at a comfortable speed, and a metronome was employed to reduce step period variability [45] and encourage a cadence between 85–90 steps per minute for consistency. The starting location of each subject was adjusted such that, on average, the center of rotation of the ankle at heel contact aligned with the rotational axis of the perturbation platform. The inter-subject average misalignment from the rotational axis was 1.75 cm ±1.56 cm, which was a small fraction of the inter-subject average of the COP range-of-motion, 21.8 cm.

Perturbations occurred in 50% of the trials to make them unpredictable. For the 2-degree study, perturbations were timed at different points after ipsilateral heel strike (100, 225, 350, or 475 ms with equal probability) to make them even more unpredictable. The 475 ms condition was excluded from our analysis because the foot occasionally lifted off the platform before the perturbation was completed and not enough time remained in the step to observe a transient response. Each set of trials had a fixed number of perturbations, where each time point was tested 10 times in a random order. The perturbation direction (dorsiflexion or plantarflexion) was chosen at random with equal probability to prevent anticipatory compensation in any one direction. Therefore, each perturbation condition (time and direction) was tested approximately 5 times per set. Ten sets were conducted for a total of 400 perturbation trials and approximately 400 unperturbed trials in the 2-degree study. We chose this large number of trials to minimize inter-subject variability, allowing us to use a small number of subjects.

Although these data were originally collected in [34] (for the purpose of estimating ankle impedance), we repeated the experiment with 5-degree perturbations to test the robustness of the phase variable hypothesis across perturbation magnitudes. After observing strong statistical significance and uniformity across perturbation times in the 2-degree experiment (see Results), the 5-degree experiment enrolled fewer subjects (seven including three repeats) and invoked only the 100 ms perturbation condition. Therefore, these experiments entailed 100 perturbed trials and approximately 100 unperturbed trials. (Note that no difference was observed between repeat subjects and new subjects in this repeated measure experiment). Data for both experiments are available from the Dryad Digital Repository: http://doi.org/10.5061/dryad.rm505.

#### Analysis

After applying a 4^th^-order Butterworth filter (20 Hz low-pass cutoff frequency) to reduce the effect of sensor noise, the recorded data was averaged across walking trials. To obtain the time-domain representation, we normalized each trial between ipsilateral heel strike and toe off (when the vertical load crossed 2% of the maximum average load). For each subject we first defined a reference timeline T of 1,000 time samples, starting at 0 ms and ending at 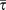, the mean time of toe off relative to initial heel strike:
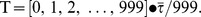



We then transformed the filtered trial data into this normalized timeline using nearest-neighbor interpolation in MATLAB. Within-subject averages for each condition were taken over the subject’s timeline.

Averaging across trials at each time sample (normalized or not) could have washed out meaningful COP variation associated with phase shifts after perturbations, so we averaged kinematic data over COP points (i.e., phase points φ) to obtain the phase domain. For each subject we defined a reference phaseline 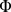 of 1,000 evenly spaced COP samples (Figure 4, right), starting at 0 m and ending at 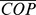, the mean value at toe off relative to the heel strike value:
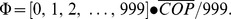



Hence, the end-points of the phaseline corresponded to the end-points of the timeline. The goal was then to define each trial’s (non-normalized) data in terms of this phaseline, e.g., the trajectory of the ankle angle *θ* was re-parameterized from non-normalized time samples (at time *t* in ms) to COP samples in phaseline Φ using nearest-neighbor interpolation, i.e.,
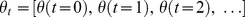
to
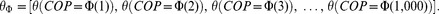



Within-subject averages for each condition were taken at each COP point in the subject’s phaseline.

Even though the time and COP ranges differed between subjects, no further interpolation was needed between the within-subject means because each subject’s timeline and phaseline were defined to be perfectly linear with even spacing. Therefore, averaging across subjects at each array index implicitly normalized the inter-subject variation. Although normalized time is often expressed as gait percentage, we plotted temporal data over a representative timeline from 0 to 0.8 s and phasic data over COP percentage (i.e., this phase variable was scaled between 0 and 100%) to avoid confusion between the temporal and feedback characterizations of phase.

#### Statistics

We performed point-wise inter-subject statistical tests over time samples for sub-hypotheses H1–H2 and over phase (i.e., COP) samples for H3–H4. Point-wise analysis is an accepted method for finding statistical deviations from normal in continuous trajectories [46], e.g., for the ankle angle. Each subject performed a large number of trials for each condition to obtain within-subject means close to the true values, which helped justify our small subject sample size: *n* = 9 for the 2-degree experiment and *n* = 7 (three repeats) for the follow-up 5-degree experiment. Our choice of a parametric statistical test, the two-sided Student’s t-test, was further justified by the Shapiro-Wilk normality test, which failed to reject the assumption that the inter-subject data came from a normal distribution. Note, however, that our results for hypotheses H0–H4 did not change when using a more conservative, non-parametric statistical test, the Wilcoxon signed-rank test. An alpha level of 0.05 was used to determine statistical significance in all tests.

## Results

To compare trials within and across subjects in the time domain, time was normalized between ipsilateral heel strike and toe off, and then trials were averaged at each time sample in a reference timeline. In order to preserve phase-dependent variability for analysis in the phase domain, we separately averaged across trials along a phaseline of evenly spaced COP samples (see Methods). This phaseline is analogous to the normalized timeline with the same number of points, so that the two domains have a unique, one-to-one correspondence during steady-state walking (but not during perturbed steps).

Sub-hypotheses H1–H4 predict a phase-dependent perturbation response in terms of the ankle deviation from nominal, which can be quite different in time domain than in phase domain if the perturbation changes the trajectory of the phase variable. For example, an increased step period after a dorsiflexive perturbation would correspond to a shallower phase trajectory (Figure 4), which we assumed when forming these hypotheses. We tested this assumption in sub-hypothesis H0 and verified that in the 2-degree experiment the step period increased with statistical significance (*p* = 0.0001) from an average of 799 ms ±26 ms in the nominal condition to an average of 815 ms ±22 ms in the 100 ms dorsiflexion condition (Table 1, and similarly for the other times in Table 2). Moreover, the step period decreased with statistical significance (*p* = 0.0008) from the nominal condition to an average of 789 ms ±30 ms in the 100 ms plantarflexion condition (and similarly for the other times, Table 2). Sub-hypothesis H0 was similarly confirmed for the 5-degree experiment. These results justify our sub-hypotheses H1–H4, which predict certain differences in the ankle deviation between the phase and time domains that would suggest phase-dependent control. Note that although the ankle angle must be the same in both domains at the end of a step, the ankle’s final deviation from nominal may still differ between domains if the perturbation changes the range-of-motion of the phase variable.

**Table 1:** Within-Subject Effect of 100 ms 2-Degree Dorsiflexive Perturbation on Stance Period.

Subject	Nominal Mean (ms)	Nominal STD (ms)	Perturbed Mean (ms)	Perturbed STD (ms)
S1	755	31	769	28
S2	825	50	845	47
S3	751	29	778	42
S4	746	33	771	43
S5	763	27	790	24
S6	785	30	807	33
S7	755	33	777	37
S8	794	35	810	34
S9	801	36	820	33

STD = standard deviation.

**Table 2:** Across-Subject Effect of 2-Degree Perturbations on Stance Period and COP Range.

Condition	Period Mean (ms)	Period STD (ms)	COP Mean (mm)	COP STD (mm)
Nominal	799	26	221	16
Dorsiflex at 100 ms	815	22	216	14
Dorsiflex at 225 ms	810	23	219	16
Dorsiflex at 350 ms	806	24	220	14
Plantarflex at 100 ms	789	30	220	17
Plantarflex at 225 ms	790	29	222	17
Plantarflex at 350 ms	792	28	221	19

COP = center of pressure, STD = standard deviation.

**Figure 5:**
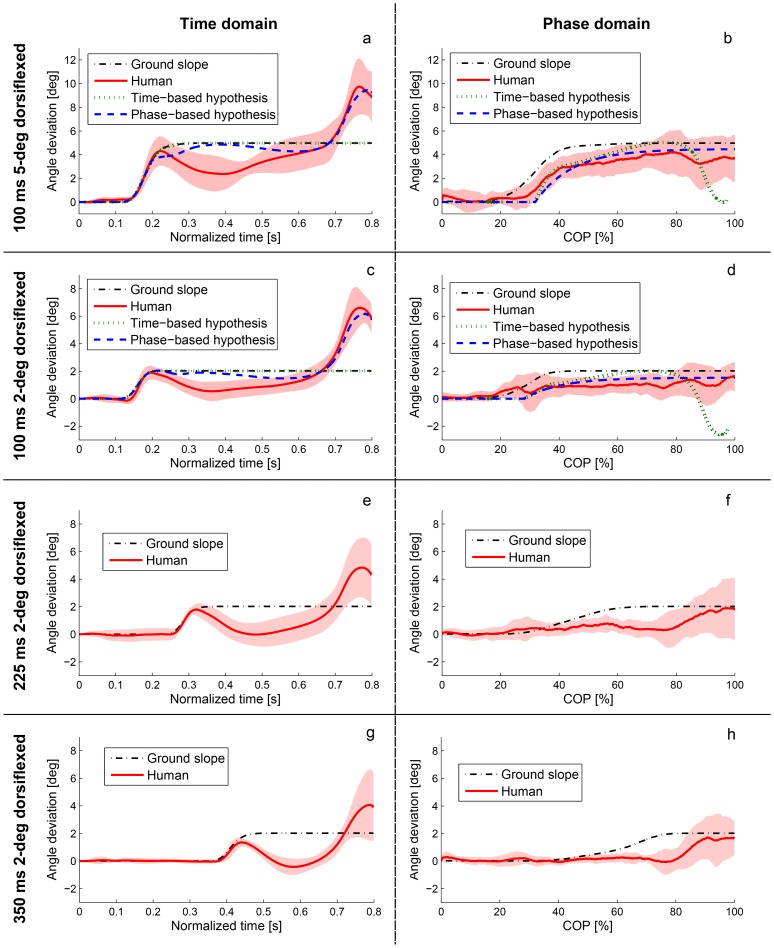
Human responses to dorsiflexive (positive) perturbations at 100 ms, 225 ms, and 350 ms after heel strike. (a–d) Human and hypothetical deviations from the nominal trajectory after 5-degree (a, b) and 2-degree (c, d) perturbations at 100 ms in both time domain (left) and phase domain (right). Human responses are shown by mean value and error bars (±1 standard deviation shown in the shaded region), and the perturbation window is shown by the ground slope trajectory in dash-dotted black. The time-based reference (dotted green) exactly follows the ground slope trajectory (dash-dotted black) in time domain, which shows the perturbation window. The phase variable hypothesis (with the COP as the phase variable) better predicts the two human cases. Note that the phase variable hypothesis is initially outside the error bars of the actual time-domain response because the hypothesis was modeled with a linear time-phase curve that did not capture the nonlinearities of the actual COP trajectory (Figure 4). The hypothetical time-phase curve does not enter into the phase-domain reference pattern, so we see that the phase variable hypothesis is well within the error bars of the actual phase-domain response. (e–h) Human deviations from the nominal trajectory after 2-degree perturbation at 225 ms (a, b) and 350 ms (c, d). The human responses to both perturbation times resemble the 100 ms response in addition to the phase variable hypothesis for the 100 ms condition (c, d). Note that the perturbation does not appear to start exactly at 225 ms in (e) or 350 ms in (g) because the slope trajectory is plotted over normalized time (which is scaled between 0 and 0.8 s). Moreover, the perturbation window appears stretched in the phase domain compared to the time domain for two reasons: 1) the COP naturally moves faster during the time window of these perturbations, which therefore occupy a larger portion of the phaseline, and 2) the COP location at perturbation times varies due to normal variation in walking speed, so averaging the perturbation trajectory over COP samples results in a widened window. In other words, perturbations start at different points in phase between trials, which are averaged into one longer window.

We begin by examining the subjects’ responses to dorsiflexive perturbations at 100 ms after heel strike. The across-subject mean trajectory of the ankle angle deviation, shown temporally in Figure 5a and 5c and phasically in Figure 5b and 5d, supports the phase variable hypothesis. The temporal response initially undershot the inclined nominal trajectory, confirming H1; this was statistically significant (*p*<0.05) between 234 and 624 ms for the 2-degree experiment and between 260 and 544 ms for the 5-degree experiment (e.g., *p* = 0.0003 and *p* = 0.0001, respectively, at 450 ms). The temporal response then overshot the inclined nominal trajectory, confirming H2; this was statistically significant after 689 ms for 2 degrees and after 712 ms for 5 degrees (e.g., *p* = 0.0000 and *p* = 0.0035, respectively, at toe off). This general behavior was predicted by the phase variable hypothesis of Figure 3c. Note that this hypothesis was initially outside the error bars of the actual response in the time domain (Figure 5a and 5c) because our hypothetical linear time-phase curve did not model the nonlinearities of the actual COP trajectory (Figure 4), i.e., the COP increased at a slower or faster rate during certain periods in the step. However, phase need not evolve linearly with respect to time [27], and we saw that the human behavior was much closer to the phase variable hypothesis in the phase domain (Figure 5b and 5d), which does not depend on temporal characteristics.

In the phase domain the perturbed ankle angle initially moved tangential to the nominal trajectory (Figure 5b and 5d), indicating a phase shift. After some delay the ankle then adapted to the new incline angle by converging towards the offset nominal trajectory. The phasic ankle deviation was not significantly different from the incline angle after 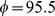
% for 2 degrees and after 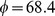
% for 5 degrees (e.g., *p* = 0.2018 and *p* = 0.1666, respectively, at toe off), confirming H3 and suggesting convergence toward the nominal inclined trajectory in phase as predicted by the phase variable hypothesis (Figure 3d). Moreover, the phasic ankle deviation was significantly greater than zero by the end of the step (*p* = 0.0056 and *p* = 0.0023, respectively), which opposes the hypothetical time-based response of Figure 3b.

Responses to dorsiflexive 2-degree perturbations at 225 ms and 350 ms, shown in Figures 5e–h, also resemble the phase variable hypothesis in both the time and phase domains. Sub-hypotheses H1–H3 were statistically confirmed for these additional conditions as described above for the 100 ms case. Hence, the phase variable hypothesis appears to hold across the stance period of gait. Because convergence took place in phase domain regardless of the timing of the 2-degree perturbations, we only tested the 100 ms time in the 5-degree repeated measure experiment.

**Figure 6:**
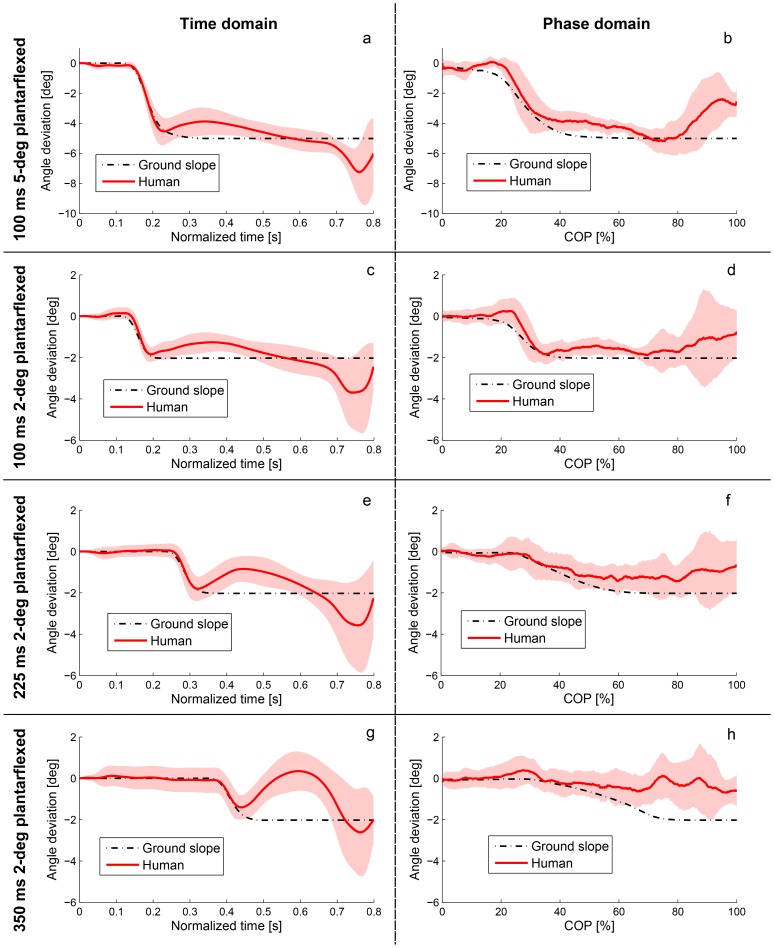
Human responses to plantarflexive (negative) perturbations at 100 ms, 225 ms, and 350 ms after heel strike. Human deviations from the nominal trajectory after 5-degree perturbations at 100 ms (a, b) and 2-degree perturbations at 100 ms (c, d), 225 ms (e, f), and 350 ms (g, h) in both time domain (left) and phase domain (right). Human responses are shown by mean value and error bars (±1 standard deviation shown in the shaded region), and the perturbation window is shown by the ground slope trajectory in dash-dotted black. The perturbed ankle trajectories appear to converge in the phase domain just below the nominal trajectory as predicted in H4 (note that humans do not normally plantarflex their ankle pattern by the slope angle during downhill walking [39]–[41], so we should not expect the ankle deviation to converge to the perturbation slope angle, see Methods). The perturbation does not appear to start exactly at 225 ms in (e) or 350 ms in (g) because the slope trajectory is plotted over normalized time (which is scaled between 0 and 0.8 s). The perturbation window appears stretched in the phase domain compared to the time domain for two reasons: 1) the COP naturally moves faster during the time window of these perturbations, which therefore occupy a larger portion of the phaseline, and 2) the COP location at perturbation times varies due to normal variation in walking speed, so averaging the perturbation trajectory over COP samples results in a widened window. In other words, perturbations start at different points in phase between trials, which are averaged into one longer window.

The plantarflexive perturbation results in Figure 6 similarly support the phase variable hypothesis. The across-subject mean ankle deviation appeared to converge in the phase domain, and not in the time domain, for both the 2-degree and 5-degree experiments. For all three timing conditions of the 2-degree study, the phasic ankle deviation remained in between zero and the incline angle for almost the entire duration of the perturbation response. In the case of the 100 ms condition, the phasic ankle deviation at 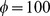
% was less than zero with *p* = 0.0403 and greater than the incline angle with *p* = 0.0095, confirming H4. In the case of 5-degree perturbations at 100 ms, the phasic ankle deviation at 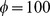
% was less than zero with *p* = 0.0005 and greater than the incline angle with *p* = 0.0005. Moreover, the temporal ankle deviation exhibited large oscillations and ended below the incline angle, suggesting that the ankle was not tracking a temporal reference in the perturbation response. These results for both perturbation directions provide evidence in support of the phase variable hypothesis.

## Discussion

Our study found that ankle responses resemble patterns produced with a time-invariant, physical embodiment of phase. The perturbation responses appeared to be primarily a feedback adaptation related to the COP with negligible time-dependent feedforward contribution. This suggests that the progression of ankle patterns during human walking is controlled by either 1) feedback related to a biomechanical phase variable instead of an internal timing variable, or 2) an internal timing variable that is *continuously* modulated by low-latency feedback related to a biomechanical phase variable. One interpretation of the first possibility is that a central pattern generator (CPG) could rely on a time-invariant, physical embodiment of phase instead of an internal timing variable when generating feedforward patterns of muscle activation. Alternatively, the observed behavior could have been produced by musculoskeletal dynamics (e.g., joint impedance [47]) with reflex mechanisms at the muscle or spinal levels using feedback related to a phase variable (e.g., adjusting the impedance equilibrium angle [34] as a function of the phase variable). State-dependent feedback unrelated to a phase variable (e.g., joint angles/velocities) must be involved in joint-level control loops to reliably track desired angles (e.g., via impedance control [47]), but the progression through the desired pattern could have depended on a single phase variable. These interpretations are consistent with evidence suggesting that time is not explicitly represented within the neural structures responsible for motor adaptation in the upper extremities [48].

A simple pattern generator that depends continuously on a biomechanical phase variable (whether through reflexes or patterns encoded in spinal centers) could easily explain phase-resetting behavior from state-dependent feedback [8], [28]. Although descending cortical drive would have to initiate this time-invariant form of pattern generator–which we call a *feedback pattern generator*–in a similar manner proposed for CPGs, a biomechanical phase variable could maintain the rhythmic pattern without an internal representation of phase. Non-rhythmic tasks like navigating extreme terrains would similarly require descending cortical drive [49].

**Figure 7:**
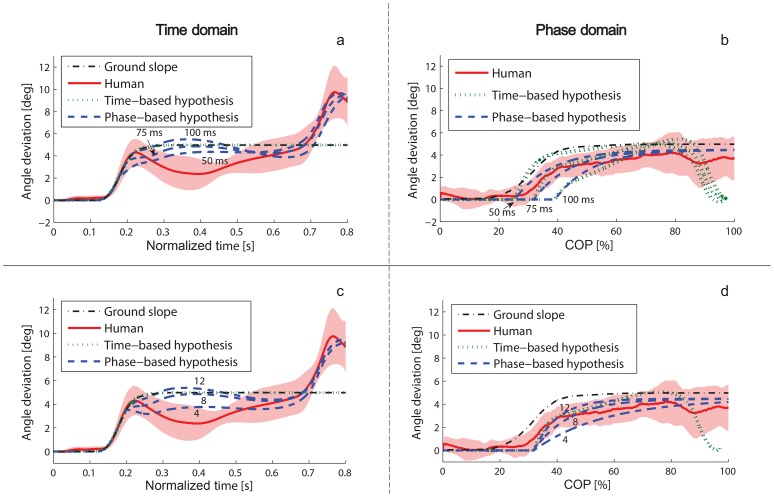
Hypothesis sensitivity to assumptions. (a, b) Human response to 5-degree perturbation vs. hypothetical responses with adaptation latencies of 50, 75, and 100 ms. (c, d) Human response to 5-degree perturbation vs. hypothetical responses with exponential convergence rates of 4·10^−4^, 8·10^−4^, and 12·10^−4^. Note that neither the latency nor convergence rate affects the time-domain response of the time-based reference because the perturbation automatically brings the ankle angle to the nominal inclined trajectory with respect to a temporal sense of phase. The two hypotheses are fairly insensitive to the latency and convergence rate assumptions, and in all cases the trend of the human response is predicted by the phase variable hypothesis.

The phase variable hypothesis is supported by similarities observed between the human perturbation response and the hypothetical phase-based response in Figure 5. This hypothetical model of the ankle response, originating in Figure 3, was justified by confirming sub-hypothesis H0 regarding the time-phase relationship (Figure 4) after both dorsiflexive and plantarflexive perturbations. Moreover, the two control strategy hypotheses are fairly insensitive to the latency and convergence rate assumptions in our hypothetical models, as we see in Figure 7 that the trend of the human response is predicted by the phase variable hypothesis for different choices of model parameters. Regardless of our hypothetical models, we observed uniformly across all perturbation magnitudes, directions, and times that the ankle deviation remained smaller in the phase domain than in the time domain (Figures 5 and 6). These results, which do not depend on any hypothetical assumptions, suggest that the ankle tracked a phase-based reference pattern in the perturbation response, which supports the phase variable hypothesis proposed in this paper.

In particular, the COP co-varied with ankle motion as would the gait cycle phase (including a phase shift in Figures 5b, 5d, 5f, and 5h as proposed in Figure 3d), suggesting that the COP may have served as a phase variable. The change in the COP trajectory after perturbations in Figure 4 was likely due to a mechanical property of the COP, i.e., the sum of moments defining the COP location [33] is affected by a change in the angle between ground and the force from gravity. The relationship observed between this COP response and the ankle response elucidates previous findings about the involvement of the COP in human locomotor control, as geometric relationships (known as “rollover shapes”) between the COP and stance leg joints are maintained across many walking conditions [31]. This strategy could be beneficial because the COP is critical to stability during walking–the location of the ground reaction force directly influences the angular momentum of the body’s center of mass [32]. A control strategy that drives ankle kinematics as a function of the COP could explain observations of positive force feedback from ankle-foot loading [28]: plantarflexive ankle torques cause forward shifts in the COP, which are in turn associated with more plantarflexion later in the step. Kinematic dependence on ankle-foot loads could also explain why foot contact is necessary to maintain kinematic coordination during body-weight supported treadmill walking [30]. This dependence is further evidenced by the reduction of ankle movement associated with altered plantar pressure distributions when walking with diabetic neuropathy [50].

The COP can be measured both from loads at the ankle and from the pressure distribution on the plantar sole [32], suggesting the involvement of a combination of load-related receptors. Similar experiments abruptly moved the ground surface during stationary standing to find that load receptors in extensor muscles activate postural reflexes [43]. Gait studies on spinal cats [51] and humans with nerve block [52] suggest that these sensory load inputs originate from Golgi tendon organs (group Ib afferents). Additionally, proprioceptive afferents from flexor muscles and cutaneous afferents detecting pressure in the plantar sole contribute jointly to postural balance control [7]. The COP corresponds to the cumulative sense of these load-related receptors involved in spinal and transcortical reflexes in humans [5], [28].

It is possible that another physical variable was employed as a measure of phase and had a one-to-one relationship with the COP, causing the relationship observed between the COP and ankle angle. In this case, the COP could be an output rather than a phase input to the human controller. Other mechanical variables that are monotonic during steady walking are phase variable candidates [27], but few variables other than the COP satisfy this property (e.g., the ankle angle does not). Another phase variable candidate is the position of the hip with respect to a global reference frame at the ankle joint (or at the COP). Grillner and Rossignol [3] alluded to this possibility after discovering that chronic spinal cats consistently initiate swing phase at the same hip angle after holding back their legs: “During the support phase, the hip joint angle increases uniformly and its value gives accurate information on how far the step has progressed.” In fact, the spinal cords of cats appear to encode sensory feedback into local representations of foot position and orientation relative to the hip [53]. The human hip angle is not perfectly monotonic during stance [33], but this variable is closely related to the global hip position, which strictly increases during steady gait. This variable is defined with respect to global axes, so measurements would require the integration of vestibular inputs across time, possibly resulting in long delays [54].

Because our study did not measure joint angles other than the ankle, we cannot conclude whether other phase variable candidates were also correlated with the ankle’s perturbation response. It is unlikely that the hip moved substantially during these perturbations due to compliance in the ankle and knee joints, in which case the hip position could not have co-varied with the ankle in a phase-locked manner. Conversely, the collected data cannot tell us whether other joints such as the knee and hip exhibited similar phase dependency on the COP. Based on observations of feedback control at the ankle and feedforward control at the hip in patients with incomplete spinal cord injury [55], we expect that distal joints including the knee may depend heavily on feedback from a phase variable, whereas proximal joints such as the hip may be controlled with a stronger feedforward component. The swing leg, being further from the COP of the stance foot, may employ a more local phase variable such as hip position, which could explain why swing foot placement appears to depend on the position and velocity of the pelvis [56]. It is also possible that the neuromuscular system combines multiple phase variables into a single quantity akin to a mathematical representation of phase [18]. Future experiments could use visual motion capture to investigate the perturbation responses of additional joints and their possible dependence on other phase variable candidates.

Our study is also limited by the exclusive use of rotational perturbations due to the platform design. By changing the ground slope, the platform perturbed both the ankle angle (our hypothesized control output) and the COP (our hypothesized control input), making it difficult to discern an input from an output in the perturbation response. The phase-locked co-variance of the ankle and COP in the response suggests the existence of a relationship, but we would have to perturb one variable and not the other to conclude whether one acts as an input and the other as an output. Moreover, rotational perturbations require us to account for two types of responses. When the robotic platform rotated the stance foot, the ankle experienced an initial dorsiflexive perturbation, followed by a constant disturbance from the subsequently inclined surface. We attempted to avoid conflating these two issues by explicitly modeling a phase shift followed by an incline change into the phase variable hypothesis based on prior studies of steady-state inclined walking [36], [39], [44]. As predicted we found that perturbations first dorsiflexed the ankle and shifted forward the COP in such a way that the ankle state moved tangential to its nominal trajectory over COP (Figure 5b, 5d, 5f, and 5h). As discussed earlier, this initial response was likely due to a mechanical property of the COP that makes it a beneficial choice of phase variable, rather than active control of the COP. Only after a delay between 50 and 75 ms did the ankle start adapting to the new incline angle by converging toward the offset nominal trajectory in phase domain (Figure 7). This inherent delay in the adaptation to the slope change allowed us to separate the two responses. Future studies could avoid the slope adaptation response by considering horizontal perturbations, which might also displace the ankle and knee without moving the COP to test whether this specific variable acts as a phase input.

Whether or not the neuromuscular system employed the COP as the actual phase variable, the COP appears to provide a good representation of gait cycle phase before and after perturbations. This property could be particularly useful in gait studies, which typically lack an observable variable that uniquely represents phase after a perturbation or stimulation [57]. The observed connection between COP and gait cycle phase also suggests that the COP could serve as an active sense of phase for controlling a prosthetic limb or humanoid robot, which currently employ statistical or adaptive techniques that integrate multiple state variables to actively estimate the phase percentage [58]. These devices often use this phase estimate or predefined rules to sequentially switch between discrete phases of the gait cycle, enacting a different control model for each [59], [60]. However, this sequential approach is not robust to phase perturbations that cause the wrong model to be used at the wrong time. The phase-based prosthetic control system in [24]–[26] avoids this problem by continuously measuring the COP to immediately respond to external perturbations or volitional movement of the subject, demonstrating the feasibility of our phase variable hypothesis in a real locomotor control system.

In conclusion, this study lays the foundation for future investigations of a novel theory of human locomotor control, wherein pattern generation might inherently depend on a time-invariant physical phase variable. Further study of our hypothesis will have important implications, from gait analysis methods to our understanding of neural control mechanisms and the subsequent design of humanoid robots [27] and lower-limb prostheses [25].

## Supporting Information

Video S1 **Video of perturbation experiment.** This video shows three consecutive trials of a perturbation experiment testing the following three conditions: 2-degree dorsiflexive perturbation, 2-degree plantarflexive perturbation, and no perturbation. The blue surface of the perturbation mechanism is a force plate. Part of the electro-goniometer measuring the stance ankle angle can be seen under the subject’s sock. When returning to the starting point for the next trial, the subject steps on a metal surface that is detached from the perturbation platform. Note that the shank and higher body segments are not moved during these perturbations, likely due to ankle compliance.
(MP4)
